# Short-Term Medical Relief Trips to Help Vulnerable Populations in Latin America. Bringing Clarity to the Scene

**DOI:** 10.3390/ijerph16050745

**Published:** 2019-03-01

**Authors:** Melodyanne Y. Cheng, Eunice Rodriguez

**Affiliations:** 1 Department of Comparative Medicine, Stanford University, Stanford, CA 94305, USA; 2 Department of Pediatrics, Stanford University, Stanford, CA 94305, USA; er23@stanford.edu

**Keywords:** short-term medical trips, international medical relief, quality of care, primary care

## Abstract

Non-profit organizations provide international medical relief trips to low/middle-income countries (LMIC) in order to provide healthcare to medically underserved areas. Short-term medical relief trips (STMRT) take a large amount of time and resources, and arouse concerns about their actual effectiveness. Here we develop a novel tool for consistently assessing how U.S. organizations provide primary care to Latin America through short-term medical relief trips. First, in Part 1, we create a “Best Practice” (BP) framework focused on the efficacy, sustainability, and long-term impact of the organizations based on a review of the last 27 years of available literature published in peer-reviewed journals. Second, in Part 2, out of 581 total medical relief organizations in the US, we identify the 19 organizations currently providing short-term primary care services to Spanish-speaking countries in Latin America. We use the BP framework to analyze the website content and secondary sources of these 19 organizations. We find that only three of the 19 organizations met 80% or more of the criteria defining BP according to the framework and four out of the 19 did not perform well in any of the framework’s three aspects of efficacy, sustainability, and long-term impact. Because there exists no current standardized way of assessing the methods implemented and services offered by STMRT, we provide suggestions about using this novel framework as a self-assessment tool for STMRT organizations.

## 1. Introduction

The United States, followed by Canada, Australia, and the United Kingdom, is estimated to send the most short-term medical relief trips to foreign countries like Mexico, Honduras, Peru, and Tanzania [1,2]. Mostly organized by non-profit organizations, private interests, and universities, short-term medical relief trips (STMRTs) often deliver either primary or specialized tertiary care to low- and middle-income countries over brief periods ranging from 1 day to 1–2 weeks [3,4]. These relief trips are a popular choice of volunteer work among college students, high schoolers, and medical professionals, with thousands of teams venturing out each year from the United States to provide free healthcare in developing countries [5,6]. These resource-expensive trips generally span about 7–10 days, or even as short as a single day, with costs conservatively estimated around $250 million [7]. All STMRT organizations researched here claimed to provide free health care to medically underserved patients in their mission statements; yet, due to evident limitations of time and resources, short-term relief trips are very restricted in what they can accomplish for patients. 

In recent years, there has been some critical discussion on the ethics of how medical relief trips operate [8,9,10,11]. Critics commonly argue that short-term medical missions act only as temporary treatments to deeper healthcare concerns, much like a Band-Aid covering a major gouge [12,13,14,15]. Improperly conducted, medical relief trips only make the host community more dependent upon an outside source [16,17,18,19,20,21,22,23]. Lack of standardized evaluation tools, ethical ambiguity when treating chronic illnesses in the communities, and limited cultural competence often recur as common concerns throughout the literature [24,25,26,27,28,29,30,31,32]. Although Maki et al. provide a basic tool to use for evaluating medical relief trips, very little interest was shown in the tool post-publication by the medical relief organizations [24]. The online tool was eventually removed from the web due to lack of use. Beyond these considerations, current measures for evaluating short-term medical relief trips completely fail to consider unintentional harm that could result from short-term medical relief trips [7,11,12,30].

The currently existing measures of evaluation of short-term medical relief trips rely primarily upon financial data tied to numbers of patients served or medical resources allocated [33,34,35,36,37,38,39,40,41,42,43]. Roughly 6000 short-term medical relief trips are sent each year, and average expenses equate about $50,000 per medical relief team per trip [24]. Short-term global medical aid efforts have gained momentum in recent years, with the considerable financial and human resources invested in this type of humanitarian aid reflecting the sheer number and popularity of short-term medical relief trips [24].

McKinsey & Company, one of the world’s most prestigious management consulting firms and an expert in measuring progress in businesses and organizations, argues that this type of quantitative data alone does not measure the real success of a non-profit organization in achieving its mission [43]. From their research on 20 leading U.S. non-profit organizations to understand the best ways to measure success in a non-profit organization, McKinsey & Company concluded that organizations need to be assessed beyond the current quantitative metrics, such as number of patients served or dollars spent per patient [43]. The current financially-incentivized methods of assessing short-term medical relief therefore fall short in terms of measuring the actual success of the STMRT organizations in fulfilling their mission statements of providing quality patient care to those in need. Outside of the World Health Organization’s published guidelines on drug and medical equipment donations, there exist no comprehensive guidelines for organizations providing international short-term medical relief [32].

Medical relief trips play an important role in providing care to patients who otherwise might not receive care. Yet, the lack of standardization and reporting for outcomes data creates a challenge for evaluating the standard of care provided during short-term medical relief trips. Much like the healthcare systems in the U.S. and other high-income countries, which emphasize evidence-based approaches to health care and bringing attention to culturally sensitive care, international medical relief organizations should strive to continually improve by addressing current limitations in the medical relief trip structure.

The overall purpose of this research paper is to investigate how short-term medical relief trip organizations operate and provide quality care to their patients, as claimed on their mission statements. Specifically, this paper focuses on international STMRT organizations serving Spanish-speaking countries in South and Central America. Our work is a first attempt to bring clarity to the current scene of medical relief and create a standardized self-assessment tool for medical relief organizations that includes both quantitative and qualitative data about quality of patient care gathered from STMRT organization websites and publicly available financial information. Yet, this study cannot be used to directly investigate what STMRT organizations are actually achieving in the field.

Here, we first develop a BP framework for assessing best practices of STMRT by operationalizing the definition of “best practice” in Part 1. In Part 2, we use the BP framework to analyze the level of best practice in all U.S. organizations currently providing primary care short-term relief trips to Latin America countries by doing content analysis of information posted in their official websites and by consulting secondary data sources to examine financial accountability/transparency.

## 2. Materials and Methods

We used a mixed methods approach. First, to develop the “best practice” framework, we conducted a rigorous qualitative analysis of peer-reviewed publications. Second, to establish the content of the “best practice” guidelines, we performed website content and financial analyses of eligible STMRT organizations. Finally, we held phone interviews to validate the BP guidelines. To first define what is considered a good practice, we developed a framework of what “best practices” would be and this framework is described in “Part 1” of the following two research methods. In “Part 2”, the second portion of our methodology section describes the methods used to select and analyze a comprehensive sample of the STMRT organizations that were working on primary care in Spanish-speaking countries in Latin America between 1990 and 2017. The Stanford IRB committee reviewed the Protocol number: 32721, and declares it to be EXEMPT.

### 2.1. Part 1: Development of a “Best Practice” Framework for Self-Assessment

**Figure 1:**
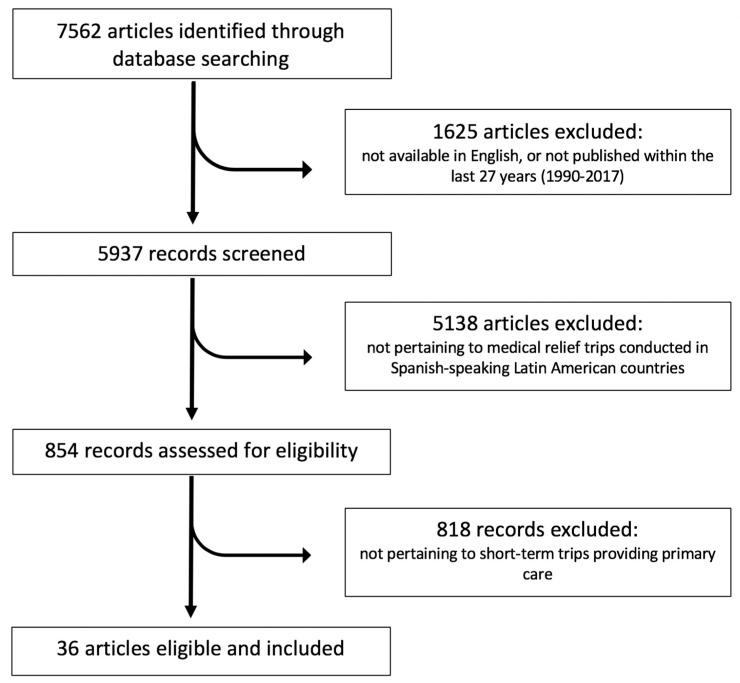
Selection of eligible articles included in literature review to identify best practices in organizations that serve Spanish-speaking countries in Latin America.

First, to develop the operationalization of a best practice definition, a rigorous literature review of all available peer-reviewed articles published in English was conducted using PubMed and the Search Works database, for which catalogs are publicly accessible on the web. We searched the following terms “medical relief trip,” “medical aid trip,” “medical mission,” “‘volunteer tourism,’” “voluntourism,” and “humanitarian aid trip” and identified 7562 unique articles. Our analysis protocol then restricted these articles to English-only review or systematic review articles published during the last 27 years (1990–2017). We screened the 5937 remaining records for articles on “medical relief trips” that were conducted in Spanish-speaking countries in Latin America. Of the remaining 854 records, we excluded out 818 articles that were not related to “short-term” medical relief trips (lasting less than 2 weeks) that provided “primary care”. We analyzed the remaining 36 articles in our literature review. This process is diagrammed below in Figure 1.

We used Dedoose, a qualitative data analysis software, to codify excerpts from the relevant literature and evaluate common themes across the 36 papers [44]. The 36 selected papers were coded according to trip aspects that pertained to before, during, and post-trip time periods using the systematic methodology of Grounded Theory, which was originally developed by Glaser and Strauss in 1967. We reviewed the data collected, and tagged repeated concepts with codes, which were extracted from the data. These codes were grouped into thematic concepts that pertained to measures or manners of evaluating the progress and outcomes of the medical relief trips and their organizations. Three independent researchers reviewed the original data and agreed upon the concepts and codes used to tag BP aspects. All concepts were finally categorized under the final 3 overarching aspects of BP were labelled as efficacy, sustainability, impact. To translate these findings into user-friendly guidelines, the conceptual measures and manners of evaluating BP were phrased as Yes/No questions under the three overarching BP aspects in a checklist format. This BP guidelines format is included in the Results section as Table 2. 

To assess the validity of the “best practice” framework, we invited all 19 non-profit organizations to participate on a phone interview, and interviewed the five organizations that accepted to participate. These open-ended interview answers were transcribed and coded using the same process described above for coding the literature review. The interview answers were tagged for repeated concepts with codes, extracted from the data. These codes were then grouped thematically into concepts and finally categorized under three overarching BP aspects. Three independent researchers participated in the coding process of the interviews. A description of the BP framework developed is included in the Results section, and a comprehensive description of the operationalized terms of efficacy, sustainability, and impact can be found in Appendix A.

The BP self-assessment framework is an instrument to assess current practices of the STMRT organizations through analysis of website content and publicly available financial information. This instrument cannot be used to assess the evaluation of the actual impact and quality of care provided. That would require direct in-field observations and it is beyond the aims of this study.

The Yes/No questions included in the BP assessment tool were created from the previously described literature review. We assessed inter-rater reliability to confirm consistency among observational ratings provided by multiple coders. Three researchers independently reviewed the original data and agreed upon the BP aspects in the guidelines. The final BP guidelines were also reviewed by two additional independent researchers, and validated by phone interviews with 5 of the 19 STMRT organizations.

### 2.2. Part 2: Selection and Analysis of STMRT Organizations

#### 2.2.1. Part 2: Sample Size Description

**Figure 2:**
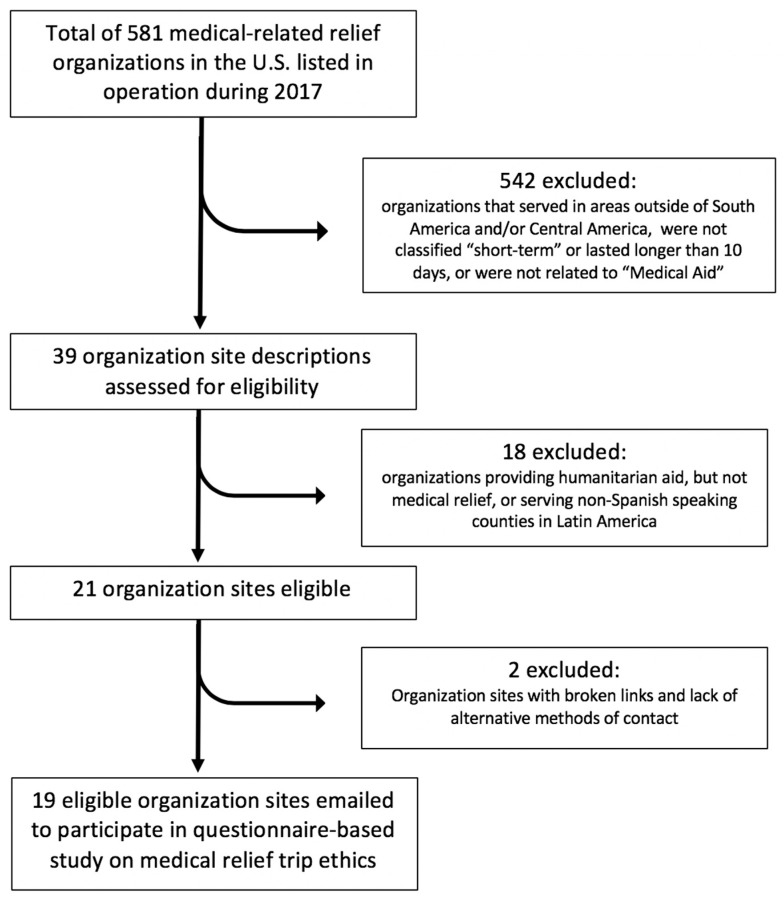
Exclusion and selection process for eligible medical relief organizations included in this research study.

To select all available and eligible organizations providing short-term primary care relief trips, we first used the two sites (MedicalMissions.com and MedicalMissions.org) that list all 581 medical-related relief trips currently in operation in the US during 2017. The process of selecting our final organizations that were both eligible and included in our study is depicted below in Figure 2. 

Then we restricted our search results to missions serving South America and/or Central America (under search field for “areas of the world”), or any of the following countries: Argentina, Bolivia, Chile, Costa Rica, Cuba, Dominican Republic, Ecuador, El Salvador, Guatemala, Honduras, Mexico, Panama, Paraguay, Peru, Uruguay, Venezuela. We further limited our search results to “Short-Term Missions” (under search field for “types of serving”), and “Medical” Aid (under search field for “healthcare specialties”). In total, there were 39 search results that fulfilled our search restrictions. Of the 39 organizations, 11 were not relevant to the research because they were not organizations hosting medical relief trips but rather other types of humanitarian health-related aid. Seven were removed from the search results because they served non-Spanish-speaking countries in Central and South America. Two sites were removed due to broken website links and/or lack of alternative contact information. The final list included the 19 relevant STMRT organizations. These organizations were contacted through email to ask if they would participate in a questionnaire-based study on medical relief trip ethics.

#### 2.2.2. Part 2: Analysis Approach

A mixed methods approach was used to analyze data from the 19 selected STMRT organizations by doing a content analysis of their websites [45]. The best practice framework, developed in Part 1 and described in the Results section, served as the basis by which to analyze the website content according to three overarching themes: efficacy, sustainability- financial transparency/accountability, and long term impact. Based off of questions about each aspect, we assessed how organizations operated in terms of best practices, such as volunteer trip preparation and resources, financial transparency/accountability, and religious affiliation and accessibility of care.

Finally, to validate the thematic BP guidelines we created in Part 1, we invited all 19 organizations to participate in confidential personal phone interviews and conducted phone interviews with the five agencies that were willing to participate. We transcribed interviews with the five medical aid organizations and used Dedoose, a qualitative data analysis software, to codify each interview transcript for common themes [44]. Three independent researchers participated in assessing the validity of this approach. Some codes included: cultural competence, efficacy, sustainability, impact, description of care provided, description of needs, description of patient communities, and religious affiliation.

## 3. Results

### 3.1. Part 1: Best Practice Framework

The analysis of the 36 articles on international medical relief trips to Latin America for primary care showed that specific themes occurred repeatedly. We divided these aspects into three time periods for analysis: pre-trip, during trip, and post-trip. Pre-trip time period covered any aspect that factor in before the medical relief trip occurred, during trip period indicates aspects that factor in while the medical relief trip is in progress, and post-trip period signifies the aspects that factor in after the medical relief trip is over.

The main aspects in the pre-trip themes included whether or not organizations checked credentials, whether or not they used foreign practitioners, whether or not they used culturally competent translators. The main aspects in the during trip themes included whether or not organizations interacted with local counterparts, whether or not they demonstrated models for growth, and whether or not they provided educational programs to improve community health. Finally, the main aspects in the post-trip themes included whether or not organizations had long-term exits plans, whether or not they prepared for post-operational consequences, and whether or not they respected cultural attitudes about health and health-giving. For the final BP guidelines format, we worded the pre-trip, during trip, and post-trip trip aspects from the literature review as yes/no questions and reorganized all the aspects into the three overarching themes of efficacy, sustainability, and long-term impact.

**Figure 3:**
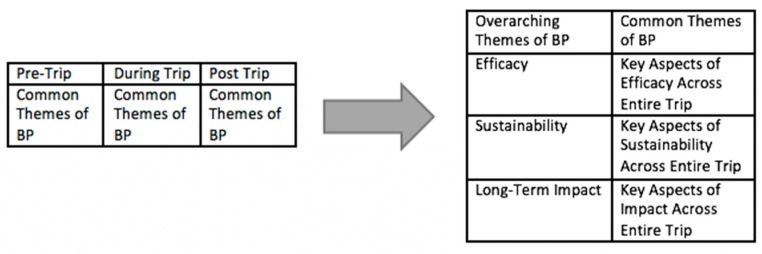
Flowchart diagram of how common Best Practices (BP) themes in literature were sorted into the 3 overarching categories of efficacy, sustainability, and long-term impact.

As previously mentioned, Figure 3 provides insight into how the framework was developed. In summary, we found there were differences in the way organizations provide and whether or not they check credentials, have orientations, recruit foreign professionals, prescribe medications, and interact with local organizations. The results combine pre-trip, during trip, and after trip best practices under three major aspects of efficacy, sustainability, and long-term impact, as demonstrated in the schematic in Figure 3. Efficacy is defined broadly in terms of pre-trip preparations, certifications, and utilization of stakeholders. Sustainability is defined in terms of financial sustainability and interactions with local communities. Long-term impact is defined in terms of end-goals, attitudes and ideology, as well as self-sufficiency. More details are described in Table 2.

### 3.2. Part 2: Description of the Sample of Short-Term Medical Relief Organizations (STMRT)

Table 1 summarizes characteristics of the STMRT. The majority of the organizations, nine out of 19, have between three and 10 staff members, while three out of 19 organizations have less than three members.

Only five of the organizations were not religiously affiliated. Of the 14 religiously affiliated organizations, the majority were non-denominational Christian, although one was Baptist and one was Catholic.

Almost all of the organizations were registered as 501(c)(3) status, classifying them as a non-profit charity. Only one organization claimed to be a non-profit organization, but was not officially registered with the governmental database of 501(c)(3) organizations. Financial transparency/accountability exists as an important measurement of STMRT organizations and will be explored further in the typology of financial transparency/accountability.

Because many of the organizations depended upon web-based methods of contact, a functional web interface was critical to accessing information about the organizations. Of the 19 organizations, one organization had an invalid web address and had to be accessed through cached storage. The majority of the organizations provided viable web interfaces.

### 3.3. Results of STMRT Organizations Content Analysis: Typology of Efficacy, Sustainability, and Impact of Medical Aid

Table 2 provides a summary of the best practices analysis of efficacy, sustainability, and impact. These three key aspects of best practice were broken down into specific questions that assessed each aspect in further detail, per each aspect’s definition.

#### 3.3.1. Efficacy

Efficacy was assessed with 11 questions covering cultural competence, resource usage, quantifiable measures of success and progress such as number of patients served, and pre-trip preparation. Only four of the 19 sampled STMRT organizations met more than 80% of common best practices assessed for efficacy. Over three-quarters, or 15 of the 19, sampled STMRT organizations met more than 30% of common best practices assessed for efficacy.

Less than half of the organizations checked the credentials of volunteer medical translators, according to their websites. Nine of the 19 organizations prepared volunteers with some pre-trip orientation or training before the actual trip send-off. About one-third, or six of the 19 organizations, prepared volunteers with some informational handouts before the medical relief trip send-off.

#### 3.3.2. Sustainability

Sustainability was assessed with 10 questions covering financial accountability/transparency, existence of advisory boards, sustainable relationships with local constituents, and programs for public health. Only three of the 19 sampled STMRT organizations met more than 80% of common best practices assessed for sustainability. Around three-quarters, or 14 of the 19 sampled STMRT organizations, met more than 30% of common best practices assessed for sustainability.

To assess financial accountability/transparency on the 19 STMRT organizations, we documented whether the organizations were registered as official 501(c)(3) organizations, which places them as official non-profit non-governmental charities. Eighteen of the 19 STMRT organizations were officially registered as 501(c)(3) organizations. We also documented whether the organizations spent more than 10% of their budget on administrative output and whether the organizations had a non-sustainable (negative) revenue. If the organizations either spent more than 10% of their budget on administrative output (three out of 19) or had a non-sustainable (negative) revenue (eight out of 19), then we marked the organization as a non-sustainable model. Eight organizations out of the 19 total were non-sustainable models of care by STMRT organizations. Only three organizations had a sustainable revenue.

#### 3.3.3. Impact

As described in Table 2, impact was assessed with 11 questions covering follow-up care and post-leave consequences, auditing, non-discriminatory policies of care, long-term exit plans, and training of local counterparts. Only three of the 19 sampled STMRT organizations met more than 80% of common best practices assessed for impact. Eight of the 19 sampled STMRT organizations met more than 30% of common best practices assessed for impact. 

To assess religious orientations and non-discriminatory policies of care by the 19 STMRT organizations, we documented whether the organizations were religious affiliated. Out of the 19 organizations’ websites sampled, 13 were religiously affiliated. Less than half, nine of the 19 organizations, received third-party accreditations. We also checked whether the organizations explicitly stated non-discriminatory policies of service; less than half, eight out of those 19 organizations, did so. Only one out of the 19 organizations actively acknowledged not imposing religious ideas on patients served.

### 3.4. Interview Results to Validate Framework

We conducted in-depth, hour-long, and open-answer interviews with 5 organizations. Our interviews clearly indicated the same three top-scoring organizations highlighted by our BP guidelines as ones that were evidently attentive to efficacy, sustainability, and long-term impact. These interviews validated the “best practices” guidelines framework that we created in this study. 

Since only five of the 19 organizations responded, we confirmed internal validity of the small sample size by checking to make sure the five organizations reflected the overall breadth of the 19 organizations in terms of religious affiliation, organization size, and other homogeneous characteristics, and we found that the five organizations had a diverse mixture of non-religious and religious affiliation among a mixture of large, medium, and small organizations.

### 3.5. Results Summary

In summary, three of the 19 organizations performed well across the board and completed 80% or more of BP for efficacy, sustainability, and long-term impact. The key elements that set these 3 organizations apart include:

- Training volunteers appropriately before the trip using handouts and a pre-trip orientation
- Utilizing local translators
- Actively minimizing resource monopolization
- Certifying credentials for both medical providers and language translators
- Maintaining a sustainable revenue
- Integrating local health systems
- Creating relationships with local practitioners/NGOs
- Preparing for post-leave medical consequences
- Planning for long-term exit
- Being able to provide long-term follow care
- Respecting and adhering to local prescription attitudes
- Helping target communities become ultimately self-sufficient without their presence
- Translating all short-term goals into long-term goals

The rest of the organizations appear to underperform in at least one or more of these three BP areas identified in the framework. Four of the organizations underperform in all three areas of BP. The 19 organizations performed better in meeting efficacy and sustainability BP than they did for long-term impact BP. More than half of the 19 organizations exhibited poor performance regarding long-term impact and completed less than 30% of BP for long-term impact.

## 4. Discussion

This work presents a first attempt to define and identify best practice and analyze the level of BP in STMRT organizations using the three identified aspects of efficacy, sustainability, and long-term impact. These results of this research present a novel and quantifiable look at the current state of practice by STMRT organizations. These results also allow us to measure the efficacy, sustainability, and impact of STMRT organizations for purposes of comparison and understanding in order to propose better policies of care and practice for future medical relief trip undertakings. Raising awareness of “best practices” by STMRT organizations could be useful in changing the standards by which STMRT currently operate and thus raise the overall quality of care.

### Limitations. Recommendations and Future Directions

This study had a number of methodological limitations. We were not able to include direct observations and we based our entire analysis on information reported by organizations and public documents. Despite those limitations, however, this study presents improvements to what has previously been done. Our methodology builds on and expands the previous work of Maki et al. assessing the quality of care given by medical relief trips [7].

An additional limitation is that we were only able to interview five of the 19 organizations. Yet, looking at the characteristics of the organization, we were reassured that they represented the breadth of the 19 organizations in the study. We had a good mix of large, medium, small organizations and a mix of non-religious and religious organizations in our sample of 19 organizations. Of the five we were able to contact personally, the organizations strongly indicated interest in exploring the final BP guidelines to work towards future self-improvement. Unlike Maki et al., we believe a clear strength of this study is our connection to our community partners, who have stayed involved in our research from start to finish and therefore may be more likely to find this self-assessment tool of interest [7]. Furthermore, we plan to share our work at public forums and conferences, with all SMR organizations, as well as researchers involved in global health at academic institutions. 

We have developed a standardized self-assessment tool to allow STMRT organizations to assess their performance. Yet, self-reported impact can be limited. In its present form, our tool cannot be used to evaluate what STMRT organizations are actually accomplishing in the field; this is a very important question that requires further investigation, and direct field observation. The immediate next step to build on this work is to improve this standardized self-assessment tool to be able to evaluate quality and quantity of short-term medical relief services in the field. It would also be useful to include more stakeholders to strengthen the validation process. In future iterations, we would consider ways to include other stakeholders, including users, policymakers, providers, in validating the BP framework.

We hope to generate enough interest to expand on this instrument and not only focus on the process itself but also add outcome measures to develop a solid evaluation tool for MR trips in general. We have developed a novel “best practices” framework with which medical relief trip organizations can self-assess their own progress and outcomes in the field. With this standardized tool for assessing the quality of care, these organizations could not only evaluate themselves against their own benchmarks, but also compare their methods, outcomes, and progress against other organizations in the field. This standardization assessment could be used to help create continuity and validity in a field that previously has not widely adopted a standardized method of assessment. This framework could also be a first step towards developing a more comprehensive evaluation protocol to assess the impact of STMRT organizations in the communities they serve. 

The three STMRT organizations that performed well across the board in meeting 80% or more of BP for efficacy, sustainability, and impact could potentially act as role models for other organizations seeking to improve. These three high-performers not only show that this improvement can and has been done in the field among a select few, but they also could suggest how to achieve such improvement and could potentially provide example models for change to other STMRT organizations.

To increase efficacy of medical relief trips, the level of cultural competency among the practitioners, and even the untrained volunteers, must be closely guarded and maintained; rather than using only student volunteers, medical missions could hire or train locals to become fully qualified medical translators. To provide continuity of care through the short-term medical relief trip model, one potential venue could be to provide consistent follow-up care through repetitive trips to the same location every few months.

To improve sustainability, most of the organizations could benefit by following the more successful models in the field. By reorganizing their organizational structure, these organizations could potentially channel more of their funding towards patient care instead of administrative overhead.

In addition, the best solution to long-term impact for a short-term medical mission is to ironically not be part of the equation any longer; in other words, the goal of a short-term medical mission should therefore be to train local medical counterparts in the host community so that they can provide an equally high level of healthcare to their community. Moreover, in the best scenario for maximal impact upon the host community, the short-term medical mission should interact fully with the local healthcare system and ensure that the working relationship is mutually beneficial; the medical mission and the healthcare system can therefore build off one another’s efforts for greatest efficacy.

## 5. Conclusions

Because the same three organizations out of the 19 total met above 80% of best practices for efficacy, sustainability, and long-term impact, there is a lot to learn from the existing infrastructure of these organizations. Through our research, we hope to increase the accountability of the medical relief trip organizations in the future. Ironically, to maximize long-term sustainability, STMRT organizations, and perhaps all medical missions in general, should work towards the eventual goal of making themselves obsolete in the developing countries that they serve.

## Figures and Tables

**Table 1:** Key characteristics of sampled short-term medical relief (STMRT) organizations.

Characteristics of STMRT Organizations	Number (and %) of STMRT Organizations
Size:	
<3 staff	3 (15.79%)
3–10 staff	9 (47.37%)
>10 staff	7 (36.84%)
Religion:	
Affiliated:	14 (73.68%)
Baptist	1 (5.26%)
Catholic	1 (5.26%)
Christian	12 (63.16%)
Non-affiliated	5 (26.32%)
Type of organization:	
501(C)(3) non-profit charity	18 (94.74%)
Not registered	1 (5.26%)
Website availability:	
Functional website	18 (94.74%)
Non-functional website	1 (5.26%)

**Table 2:** Summary of best practices (BP) and breakdown of organizations meeting specific BP thresholds.

Aspects of Best Practices (BP)	Specific Questions Assessed Within Each Aspect *	# Organizations Meeting	% of Organizations Meeting Best Practices (BP)		
			80–100% BP	30–79% BP	0–29% BP
Efficacy	Acknowledges importance of cultural competence/humility?	5	21.05% (4)	57.89% (11)	21.05% (4)
	Actively minimizes resource monopolization?	2			
	Application necessary to join?	19			
	Certification of practitioner credentials?	19			
	Certification of translator abilities?	9			
	Medical record system for all patients?	12			
	Orientation of volunteers before trips?	9			
	Resources/handouts available to educate volunteers on website?	6			
	Tracks how much funding goes directly toward patient services?	6			
	Tracks number of patients served?	19			
	Utilization of local translators?	3			
Sustainability	Board of directors/expert advisors in addition to leadership team?	13	15.79% (3)	57.89% (11)	26.32% (5)
	Disclosure of financial statements on website?	6			
	Integration of local health systems?	3			
	Minimizes expenditure on administration budget? (measured as <10% of total revenue)	16			
	Programs on community health and/or public health?	7			
	Registered as official 501(c)(3) charity/non-profit?	18			
	Sustainable (positive) revenue?	11			
	Transparent executive statements of funding diagrams available on website?	6			
	Working relationships with local medical practitioners?	2			
	Working relationships with local NGOs?	2			
Impact	Ability to provide long-term follow-up care?	4	15.79% (3)	26.32% (5)	57.89% (11)
	Accredited by third-party audits?	9			
	Actively acknowledges not imposing religious ideas on patients served?	1			
	Explicitly states non-discriminatory policy or service?	8			
	Plans for long-term exit?	3			
	Plans for sustainable and independent local healthcare?				
	Preparation for post-leave medical consequences?	3			
	Respect and/or adherence for local-based prescription attitudes?	2			
	Sustainability/long-term impact mentioned in mission statement?	8			
	Training of local medical and non-medical counterparts?	6			
	Translation of all short-term goals into long-term goals?	3			

* Definitions for each question assessed are provided in Table A1 under Appendix A.

## Appendix A

### Appendix A.1. Operationalizing Efficacy, Sustainability, and Long-Term Impact of STMRT Organizations

For efficacy, we operationalized whether the 19 STMRT organizations checked credentials by asking whether the STMRT’s website asked to verify volunteer medical professionals’ credentials through some application or other method of authentication. We also operationalized whether these organizations provided informational handouts as a form of volunteer preparation during the pre-trip period by asking whether the STMRT’s website mentions existence of some type of informational/guiding hand-outs prior to the trip send-off. Finally, we operationalized whether the 19 STMRT organizations engaged in volunteer training prior to trip send-off by asking whether the STMRT organization website mention requirements or existence of a pre-trip volunteer training or some type of volunteer orientation prior to the trip send-off.

For sustainability, we evaluated the numerical breakdown of financial accountability/transparency of 19 STMRT organizations. We first operationalized the category of status as 501(c)(3) non-profit organization by verifying each organization’s stated status against verified status checkers available on GuideStar, a website based at guidestar.org that provides the world’s largest source of information about non-profit organizations. We also checked the status against the organization’s last released public IRS tax forms, Form 990, which confirms the status of the organization as a non-profit organization because only charities and non-profits can file IRS Form 990. We operationalized the category of receiving third-party accreditations by searching GuideStar for listed accreditations for each organization, as well as referencing the organization’s website itself. All accredited organizations placed logos on their webpage near the donation page or the front page in order to inspire confidence in potential donors that their organizations were trustworthy enough to merit the donated dollars. We operationalized the last two categories, sustainable (positive) revenue, and spending less than 10% of budget on administrative costs, by checking released IRS tax forms (Form 990) for each of the 19 STMRT organizations. Additionally, we searched the organizations’ websites for sections on financial accountability/transparency; most of the larger, more upfront sites provided their released tax forms directly on the site in an attempt to inspire confidence in their finance-budgeting abilities and trustworthiness. Some sites even created executive summaries and helpful data figures to illustrate the breakdown of their spent finances during the most recent year.

For impact, we operationalized the category of religiously affiliated by searching through each organization’s website for any mentions of faith or faith-based practice. We also operationalized the category of explicitly stated non-discriminatory policy of care by asking whether STMRTs explicitly state anywhere in their websites that they are equally willing to provide the same quality and level of care to patients that may be of a different religion, culture, or belief system. We operationalized the category of active acknowledgement of not imposing ideas on patients served by asking the following question. Does this STMRT organization explicitly acknowledge anywhere in its website that it will not impose or force ideas (religious-based) on patients served during the trip by any of its volunteers?

**Table A1:** Definition for Y/N Questions Assessing BP in Table 2.

Aspects of Best Practices (BP)	Specific Questions Assessed Within Each Aspect *	Definition
Efficacy	Acknowledges importance of cultural competence/humility?	Website mentions cultural competence/humility or respect for patient culture
	Actively minimizes resource monopolization?	Actively minimizes resource usage per patient (in terms of financial expenditure and medical supplies); posted on websites
	Application necessary to join?	Website contains application or information on how to apply with qualifications
	Certification of practitioner credentials?	Website states organization verifies volunteer medical professionals’ credentials through some application or other method of authentication
	Certification of translator abilities?	Website provides application or information on how to apply with qualifications for medical translation
	Medical record system for all patients?	Website mentions a medical record system in place for patients served or alternative method of keeping track of returning patient history
	Orientation of volunteers before trips?	Whether the STMRT organization website mention requirements or existence of a pre-trip volunteer training or some type of volunteer orientation prior to the trip send-off
	Resources/handouts available to educate volunteers on website?	Whether the STMRT’s website mentions existence of some type of informational/guiding hand-outs prior to the trip send-off
	Tracks how much funding goes directly toward patient services?	Website states how much of total money raised is spent on patients
	Tracks number of patients served?	Website states how many patients are served
	Utilization of local translators?	Website states local translators are recruited and/or trained
Sustainability	Board of directors/expert advisors in addition to leadership team?	Website states existence of board of directors/expert advisors
	Disclosure of financial statements on website?	Website discloses financial statements
	Integration of local health systems?	Website states collaboration/integration with local health systems, or manner or having checked for the existence of local medical services in their chosen communities
	Minimizes expenditure on administration budget? (measured as <10% of total revenue)	Administrative budget information from released IRS tax forms (Form 990) for each of the 19 STMRT organizations
	Programs on community health and/or public health?	Website states interaction with and/or creation of community/public health programs
	Registered as official 501(c)(3) charity/non-profit?	Verified through GuideStar, a website based at guidestar.org that provides the world’s largest source of information about non-profit organizations and organization’s last released public IRS Form 990
	Sustainable (positive) revenue?	Checked against released IRS tax forms (Form 990) by subtracting total expenditures (i.e., administrative costs) from total revenue (i.e., donations)
	Transparent executive statements of funding diagrams available on website?	Website provides transparent executive statements on funding
	Working relationships with local medical practitioners?	Website states working relationships with local practitioners
	Working relationships with local NGOs?	Website states working relationships with local NGOs
Impact	Ability to provide long-term follow-up care?	Website states method of long-term follow-up care
	Accredited by third-party audits?	Verified through GuideStar by searching listed accreditations for each organization, as well as referencing the organization’s website itself
	Actively acknowledges not imposing religious ideas on patients served?	Website explicitly acknowledges that organization will not impose or force ideas (religious-based) on patients served during the trip by any of its volunteers
	Explicitly states non-discriminatory policy or service?	Website states organization is equally willing to provide the same quality and level of care to patients that may be of a different religion, culture, or belief system
	Plans for long-term exit?	Website mentions idea of long-term exit
	Plans for sustainable and independent local healthcare?	Website mentions helping local healthcare system to become sustainable and independent
	Preparation for post-leave medical consequences?	Website states plans/preparation to deal with post-leave medical consequences
	Respect and/or adherence for local-based prescription attitudes?	Website states organization respects/adheres to local-based prescription attitudes
	Sustainability/long-term impact mentioned in mission statement?	Website mentions sustainability/long-term impact ideas in mission statement
	Training of local medical and non-medical counterparts?	Website mentions training of local counterparts
	Translation of all short-term goals into long-term goals?	Website states eventual long-term goals
